# Towards Quantifying the Uncertainty in Estimating Observed Scaling Rates

**DOI:** 10.1029/2022GL099138

**Published:** 2022-06-18

**Authors:** Haider Ali, Hayley J. Fowler, David Pritchard, Geert Lenderink, Stephen Blenkinsop, Elizabeth Lewis

**Affiliations:** ^1^ School of Engineering Newcastle University Newcastle upon Tyne UK; ^2^ Royal Netherlands Meteorological Institute De Bilt The Netherlands

**Keywords:** rain‐gauge data, extreme precipitation, dewpoint temperature, quality‐control, scaling, observed precipitation

## Abstract

Short‐duration precipitation extremes (PE) increase at a rate of around 7%/K explained by the Clausius‐Clapeyron relationship. Previous studies show uncertainty in the extreme precipitation‐temperature relationship (scaling) due to various thermodynamic/dynamic factors. Here, we show that uncertainty may arise from the choice of data and methods. Using hourly precipitation (PPT) and daily dewpoint temperature (DPT) across 2,905 locations over the United States, we found higher scaling for quality‐controlled data, all locations showing positive (median 6.2%/K) scaling, as compared to raw data showing positive (median 5.3%/K) scaling over 97.5% of locations. We found higher scaling for higher measurement precision of PPT (0.25 mm: median 7.8%/K; 2.54 mm: median 6.6%/K). The method that removes seasonality in PPT and DPT gives higher (with seasonality: median 6.2%/K; without seasonality: median 7.2%/K) scaling. Our results demonstrate the importance of quality‐controlled, high‐precision observations and robust methods in estimating accurate scaling for a better understanding of PE change with warming.

## Key Points

- We estimated scaling rates from hourly observed precipitation and daily dewpoint temperature over the conterminous continental United States
- We highlight the importance of quality‐controlled, high precision observations and robust statistical methods in estimating accurate scaling
- Our results have implications for a better understanding of change in extreme precipitation and flash floods with warming

## 1 Introduction

Short‐duration precipitation extremes are intensifying with global warming and they have strong impacts on flash floods, landslides and debris flow (Fowler, Ali, et al., 2021; Fowler, Lenderink, et al., 2021; Fowler, Wasko, et al., 2021). They are particularly damaging as, within a short period, they cause severe socio‐economic impacts to urban infrastructure, disruption to transportation and loss of life (Fadhel et al., 2018). Therefore, decision‐makers need to understand potential changes to their frequencies and intensities for effective risk assessments and climate adaptation. One method used is to study the past response of precipitation extremes to temperature and then project their future changes with different levels of warming (Ali & Mishra, 2018b). However, this is not straightforward. The Clausius‐Clapeyron (CC) relationship has been used as a benchmark to interpret the changes in extreme precipitation with warming (O’Gorman, 2015). Here, warmer air is associated with higher saturation vapor pressure, holding more moisture and thus leading to intense precipitation. This “scaling” relationship links air temperature and atmospheric humidity and states that under the limitation of constant relative humidity, atmospheric moisture will increase at a rate similar to the dependency of vapor pressure on temperature (the CC rate: 6%–7%/K) (Trenberth et al., 2003). This theory is supported by scaling studies, which have estimated relationships between daily/sub‐daily precipitation intensities and day‐to‐day temperature variability – the apparent scaling – and reported scaling rates at around CC for many regions and globally (Ali et al., 2018; Ali, Fowler, et al., 2021; Ali, Peleg, et al., 2021; Bao et al., 2017; Gao et al., 2018; Martinkova & Kysely, 2020; Wasko et al., 2016).

However, many studies have also reported a deviation from CC scaling, and this varies considerably across different regions and seasons (Ali et al., 2018; Zhang et al., 2017). One of the most important factors for this deviation is moisture limitations at higher temperatures and the choice of the temperature scaling variable (Lenderink et al., 2018). For instance, using surface air temperature (SAT) as the scaling variable produces a peak structured scaling curve showing an increase in extreme precipitation up to a certain SAT threshold and then a monotonous decrease at higher SATs (Ali & Mishra, 2017; Hardwick Jones et al., 2010; Wang et al., 2017). There have been robust discussions on the cause of this, ranging from the instantaneous cooling effect of extreme precipitation (Bao et al., 2017), synoptic variability (Barbero et al., 2018) or a decrease in relative humidity at higher temperatures (Gao et al., 2020; Lenderink et al., 2018). This implies that the CC relationship may not be followed at high temperatures, since relative humidity is not constant. To overcome these humidity limitations, dewpoint temperature (DPT) has been proposed to estimate extreme precipitation‐temperature sensitivities; this captures changes to both relative humidity and SAT (Ali & Mishra, 2017; Barbero et al., 2017; Lenderink & Attema, 2015). Since DPT is the temperature at which a parcel of air reaches saturation (100% relative humidity) when cooled adiabatically, a one‐degree rise in DPT is equivalent to a 7% rise in atmospheric moisture content (Lenderink & van Meijgaard, 2008). Ali, Fowler, et al., 2021 showed a consistent global scaling of hourly precipitation at the CC rate by using DPT as the scaling variable.

Other reasons for deviation in scaling have been covered extensively elsewhere (Fowler, Ali, et al., 2021). These may be due to drier surface conditions at high temperatures (Trenberth & Shea, 2005), temperature seasonality (Ali et al., 2018), mixing of different rainfall types (Molnar et al., 2015), weather types (Blenkinsop et al., 2015; Magan et al., 2020), the intermittent nature of precipitation (Visser et al., 2020) and even inappropriate modeling assumptions (Pumo et al., 2019; Wasko et al., 2015). Scaling rates can be further changed by considering the role of large‐scale circulation patterns, deviations in local moisture availability through upward motions and moisture convergence, and local‐scale dynamics (Ali & Mishra, 2018a; Guerreiro et al., 2018; Lenderink et al., 2017; Pfahl et al., 2017), although this is not well understood (Fowler, Ali, et al., 2021; Fowler, Lenderink, et al., 2021).

Uncertainty in scaling estimates may also arise from the choice of data. Some previous global scaling studies (e.g., Moustakis et al., 2020; Wasko et al., 2016) have used reanalyses, satellite or climate model outputs to estimate precipitation, due to limitations in the availability of sub‐daily observations. Recently, Ali, Peleg, et al. (2021) checked the performance of reanalyses (ERA5 and MERRA2) against observations for different macro‐regions, finding an underestimation of hourly scaling rates for the reanalyses compared to gauge observations. Their results question the reliability of using reanalysis products. In addition to the choice of data, different scaling methods (based on different statistical methods and assumptions) may lead to uncertainty in the scaling estimate. These important considerations have been addressed previously; thus scaling studies are often not easily comparable, using different metrics of “extremeness”, different datasets, different statistical methods and scaling variables, and often lacking quality control (Fowler, Lenderink, et al., 2021). Fowler, Lenderink, et al. (2021) suggested that a “full comparison of existing methods—a meta‐analysis on scaling—would provide information on the consistency of scaling across space and whether, and in which cases/regions, this is a likely candidate to explain and predict future changes to extreme precipitation from warming.” This should use a long‐term, quality‐controlled sub‐daily gauge observations data set (Fowler, Lenderink, et al., 2021). One such example is the Global Sub‐daily Rainfall (GSDR) data set (Lewis et al., 2019), developed after extensive quality control, as discussed in Lewis et al. (2021).

In this paper, we use only hourly precipitation (PPT) gauge observations over the conterminous continental United States (CONUS), pairing these with daily DPT measurements from station observations to narrow the uncertainty in scaling rates which can arise due to data quality control and measurement precision. We limited this study to the US (as an example) because the US covers different climate zones and has a longer duration of the data at different (2.54 and 0.25 mm) measurement precisions as compared to other macro‐regions where the hourly PPT gauge observations are available (discussed in Ali, Fowler, et al., 2021). We also assess the effect of different statistical assumptions and scaling methods on the scaling estimates. In Section 2, we detail the data used and the methods investigated. In Section 3, we then present the scaling results for differences in quality control, measurement precision and statistical methods. Finally, in Section 4 we provide our conclusions and recommendations for methodological and data choices to provide more robust scaling estimates.

## 2 Data and Methods

### 2.1 Precipitation and Dewpoint Temperature Data

We use hourly precipitation observations (PPT) from two datasets across 2,905 locations over CONUS. The first originally downloaded from NOAA/NCDC (RAW‐DATA), has some initial quality control, according to their website. The second (GSDR‐QC) is the same set of gauge observations from the Global Sub‐Daily Rainfall (GSDR) data set (Lewis et al., 2019, 2021) and their quality control is discussed in the supplemental information. This data set was generated under the Global Water and Energy Exchanges (GEWEX) Hydroclimateology Panel INTENSE project (Blenkinsop et al., 2018) and has been used in many studies (Ali, Fowler, et al., 2021; Ali, Peleg, et al., 2021; Barbero et al., 2017, 2019; Li et al., 2020). We compare these (GSDR‐QC) to the same hourly precipitation observations (RAW‐DATA) over CONUS.

We use daily dewpoint temperature (DPT) from the Met Office Hadley Center observations data set: HadISD (version 2.0.2.2017f) (Dunn, 2019). This data set is available from 1/1/1931 to 12/12/2017 and comprises 8103 stations globally based on the Integrated Surface Data set (ISD) from the National Oceanic and Atmospheric Administration's (NOAA's) National Climatic Data Center (NCDC).

Since the GSDR and HadISD stations are not at the same location, for each PPT station, we identified the nearest DPT station, requiring that the distance between PPT and DPT stations is less than 40 km and their elevation difference is less than 50 m (Figure S1 in Supporting Information S1). Then we matched hourly PPT to the corresponding DPT values, ensuring that any station selected in our study has at least 12 years of PPT‐DPT pairs. This pairing process is discussed in detail in Ali, Fowler, et al. (2021). Furthermore, to check for any bias in scaling estimates due to record length, we pooled PPT‐DPT pairs from 3 to 5 neighboring locations (with a distance less than 40 km).

### 2.2 Methods

We used three methods for estimating scaling: (a) Binning Method (Lenderink & Van Meijgaard, 2008), (b) Quantile Regression (Wasko & Sharma, 2014), and (c) Zhang et al. (2017) method (ZM). These are discussed in detail in Ali, Fowler, et al. (2021).

To summarize, in BM, we first considered all wet hours (with precipitation ≥2.5 mm) for each station's PPT‐DPT pair and then distributed data into 12 (20) bins of equal size, sorted from the lowest to highest DPT. We then estimated the 99th percentile of PPT (P99) and the mean DPT for each temperature bin. We excluded the first and last bins while estimating scaling rates to avoid any absurd DPT values (outliers to climatology) arising from very specific circulations (as Ali, Fowler, et al., 2021). We fitted a linear regression on the logarithm of P99 and mean DPT using two cases (a) from the second bin to the second last bin (2 to 2last) and (b) from the second bin to the bin (breakpoint; BP) where the maximum of P99 occurred (2 to BP), denoted by:

(1) log(P99)=α+βT

Then the scaling (dP99 (%)/K) was estimated using an exponential transformation of the regression coefficient (*β*) given by:

(2) $$dP99\left(\%\right)/K=100\left(e^{\beta }-1\right)$$

QR is similar to BM except there is no assumption of the number and size of bins (Wasko & Sharma, 2014). In QR regression the best fit of a quantile to the full data set is computed assuming a linear relation. We again used Equations 1 and 2 to estimate scaling rates using all wet hours for paired PPT‐DPT data for P99 in the QR method.

The Zhang et al. (2017) method tries to overcome the role of seasonal trends in DPT in dominating the increase in extreme precipitation with DPT, by removing the seasonality from the DPT data to estimate the scaling. In the ZM method, we first identify the 4 months (Monsoon season (June‐September) for the CONUS) for each location that receives the highest hourly PPT in a year and then estimate the DPT anomalies (compared to the monthly means) for these 4 months. For the selected 4 months, we then normalize the maximum hourly PPT value (in these 4 months) by the median of the maximum hourly PPT (obtained from the whole hourly time series for the selected 4 months). We further fit a Generalized Extreme Value distribution (GEV) to these normalized maximum hourly PPT data using the corresponding DPT anomalies as a covariate on the location parameter in the GEV model. This method has been discussed in detail in Ali et al. (2018) and Ali, Fowler, et al. (2021).

## 3 Results and Discussion

### 3.1 Scaling Rates From Different Versions of Quality‐Controlled Precipitation Data

**Figure 1:**
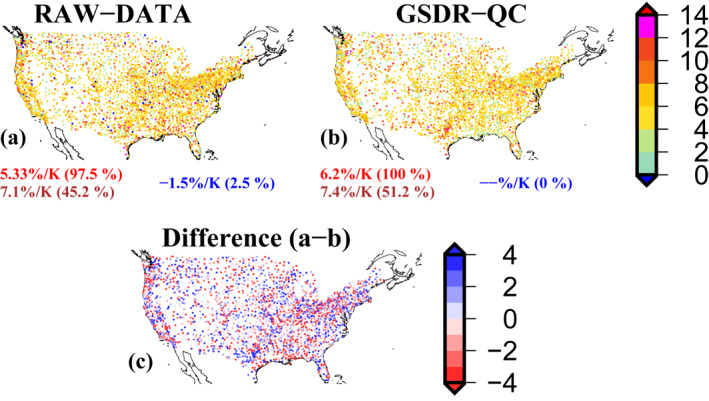
Scaling rates (% K^−1^) were estimated using hourly precipitation (PPT) from (a) NOAA/NCDC observations with initial quality control (RAW‐DATA), (b) a quality‐controlled (GSDR‐QC) version of the Global Sub‐daily Rainfall (GSDR) data set (Lewis et al., 2021), and daily dewpoint temperature (DPT) from the HadISD data set (Dunn, 2019), (c) scaling difference (a–b; % K^−1^) using RAW‐DATA and GSDR‐QC versions of data. Scaling is estimated using the binning method at the 99th percentile from the second to the second last bin (2 to 2last) for 2,905 gauges, which have at least 12 years of hourly precipitation data. The numbers below each panel show positive (red), super‐CC (brown) and negative (blue) median scaling rates respectively (numbers in parentheses show the percentage of locations with positive/super‐CC/negative scaling respectively). This figure and subsequent figures were plotted using the Generic Mapping Tool (GMT).

We estimate scaling rates for the two versions of the CONUS data set from NOAA/NCDC (RAW‐DATA) and the quality‐controlled GSDR data (GSDR‐QC). Here we estimate scaling using the binning method at the 99th percentile from the second to the second last bin (2to2last) for 2905 gauges, although results are consistent for other (QR/ZM) methods of estimating scaling. We find a considerably lower median of scaling rates over the CONUS using RAW‐DATA as compared to the GSDR‐QC data (Figure 1c). Using RAW‐DATA, around 2.5% (from 2905) of gauges show negative scaling, 97.5% of gauges show (median 5.33%/K) positive scaling and around 45% of gauges show super‐CC (higher than CC) scaling (median 7.1%/K) (Figure 1a). However, after quality control with the GSDR‐QC, all gauges show positive scaling (median greater than 6%/K) and more than half of the gauges show super‐CC scaling (median scaling 7.4%/K). Our results highlight that without extensive QC procedures the scaling rates can be substantially affected (Figure 1).

**Figure 2:**
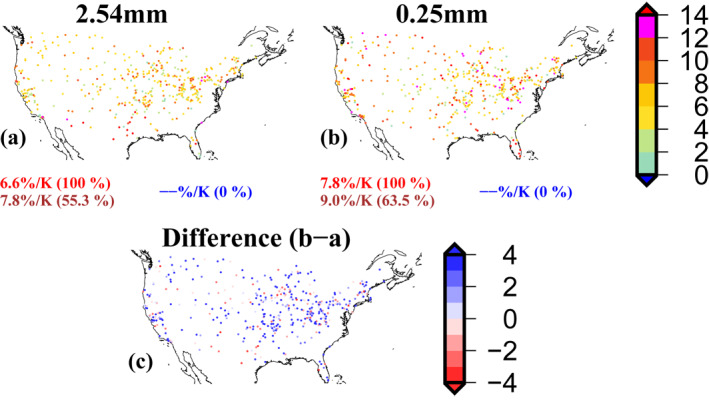
Scaling rates (% K^−1^) were estimated using hourly precipitation (PPT) from the updated version of Global Sub‐daily Rainfall (GSDR) (V2; Lewis et al.(2021), under preparation) at (a) 2.54 mm precision and (b) 0.25 mm precision, (c) scaling difference (b–a; % K^−1^) using 0.25 and 2.54 mm precision data. Scaling is estimated using the binning method at the 99th percentile from the second to the second last bin (2 to 2last) for 475 gauges that have at least 7 years of PPT data. The numbers below each panel show positive (red), super‐CC (brown) and negative (blue) median scaling respectively (numbers in parentheses show the percentage of locations with positive/super‐CC/negative scaling respectively).

We explore the reasons for the differences in scaling rates between these two versions of the CONUS hourly precipitation gauge data set by examining a few gauges in detail. We identified three stations (US_116605, US_914229 and US_020768) with the highest scaling difference from two versions of the data, arising from the errors in precipitation values in the RAW‐DATA. For example, for station US_116605, the very high precipitation values like 508 mm/hr (exceeding the world record hourly precipitation, that is, 401 mm/hr) and 251.46 mm/hr reported in the RAW‐DATA are removed in the GSDR‐QC version as they are possible units or transcription errors (Figure S2 in Supporting Information S1). Moreover, for stations: US_914229 and US_020768, the scaling differences arise due to the removal of data period having doubtful precipitation values based on the multiple checks as discussed in Supporting Information S1.

### 3.2 Scaling Rates Using Different Measurement Precision of Precipitation Data

The CONUS PPT gauges from NOAA/NCDC used in the GSDR data set are of mixed‐measurement precision (0.25 and 2.54 mm respectively). To produce a consistent measurement precision, all gauges in the CONUS were explicitly processed to have a consistent 2.54 mm precision in previous studies using the GSDR data (Ali, Fowler, et al., 2021; Ali, Peleg, et al., 2021; Barbero et al., 2018, 2019). During this conversion process, all of the small hourly precipitation amounts (less than 2.54 mm) were accumulated until they reached 2.54 mm precipitation; therefore, precipitation of at least 2.54 mm was assigned to that hour and zero precipitation was assigned to preceding hours (a procedure developed by Groisman et al., 2012). This affects the number of wet hours that appear in 2.54 mm precision GSDR data and can influence analyses that use precipitation thresholds to determine wet hours, such as scaling estimation or trend analysis (Wasko et al., 2022).

Here, we explore the limitations of using coarser precision gauge data in estimating scaling. Given the limitations in the data records of 0.25 mm precision, to have a sufficient number of gauges for the comparison, we selected gauges (475 in total) with at least 7 years of data. We acknowledge that this is not of sufficient duration to estimate robust scaling rates. Notwithstanding this limitation, we compare scaling rates over the CONUS using two different measurement precisions – 0.25 and 2.54 mm over these 475 locations (where both data exist), with the same thresholds set to define a wet hour respectively. We find that scaling rates are higher for higher measurement precision data (0.25 mm: median 7.8%/K; 2.54 mm: median 6.6%/K), with the number of gauges showing super‐CC scaling higher for the higher measurement precision data (Figure 2). Some of the scaling differences may result from (a) an increased number of PPT‐DPT pairs for the 0.25 mm precision data, and (b) higher precipitation values which are lost (smoothened over time) during aggregation from 0.25 to 2.54 mm conversion, giving a more robust estimates. Our results highlight the importance of measurement precision of rainfall gauges in scaling estimates, which is generally ignored due to the limitations in the availability of observational data.

### 3.3 Scaling Rates From Different Statistical Methods

**Figure 3:**
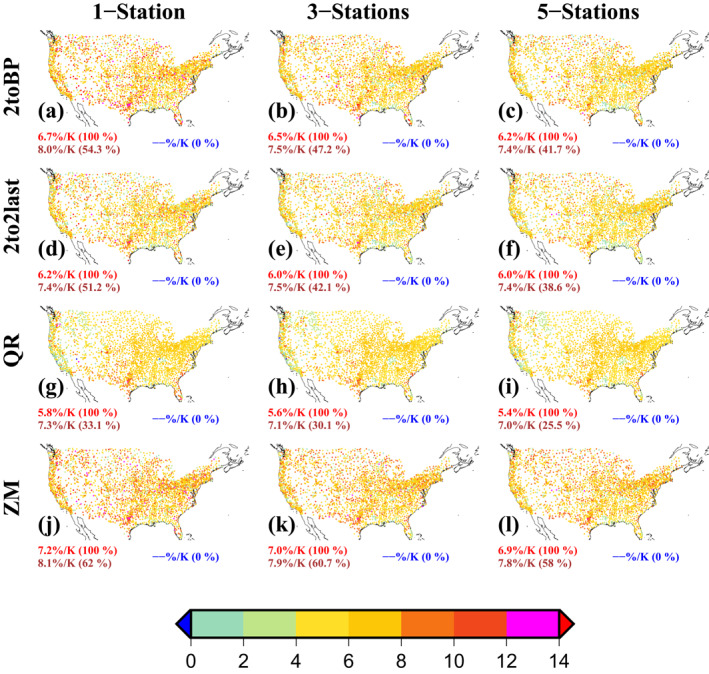
Scaling rates (% K^−1^) were estimated using hourly precipitation (PPT) from the Global Sub‐daily Rainfall (GSDR) data set (Lewis et al., 2021; 2.54 mm precision) and daily dewpoint temperature (DPT) from the HadISD data set (Dunn, 2019). Scaling is estimated using the second bin to the breakpoint (2 to BP) in the binning method (first row), second bin to the second last bin (2 to 2last) in the binning method (second row), quantile regression (QR; third row) and Zhang et al. (2017) method (ZM; fourth row) respectively. The scaling has been estimated at an individual location (1‐Station; first column), pooling three nearest locations (3‐Stations; second column), and pooling five nearest locations (5‐Stations; third column) respectively. The numbers below each panel show positive (red), super‐CC (brown) and negative (blue) median scaling rates respectively (numbers in parentheses show the percentage of gauges with positive/super‐CC/negative scaling respectively).

We now estimate scaling rates using different statistical methods: BT, QR and ZM. We find that median scaling rates over the CONUS are at least CC for BM and ZM methods, but not for QR (Figure 3). We find the highest scaling rates using ZM and the lowest using QR. More than half of the gauges show super‐CC scaling (median 8%/K) using the BT (2toBP) and ZM methods. We also pool the three and five nearest stations together to increase the number of PPT‐DPT pairs to estimate scaling to check the consistency of our results. Our results show only a slight decline in median scaling rates when more gauges are pooled together, with the same differences found between methods.

Differences between scaling rates from the different statistical methods can be large. For instance, a difference in scaling rates between two methods of binning can occur if the breakpoint (BP: the point where a maximum precipitation intensity occurs) doesn't lie near the end bin (the maximum temperatures); then scaling is only estimated up to the BP without utilizing the entire DPT range. Similarly, as the QR method accounts for all PPT‐DPT pairs, outliers in DPT associated with climatology cause lower scaling rates. Interestingly, results for the ZM method show the importance of removing seasonality in estimating scaling rates which is ignored in most scaling studies and is not considered in the other statistical methods. Our results show that scaling rates can be highly sensitive to the statistical method used; hence differences in scaling rates between studies may be a statistical artifact. This highlights the importance of using robust and consistent methods in estimating scaling rates.

## 4 Discussion and Conclusions

In this study, we examined how the choice of data and statistical methods can influence the estimation of scaling rates. Gauge observations are the best option for scaling studies since other precipitation datasets like satellite products (Wasko et al., 2016) and reanalyzes (Ali, Peleg, et al., 2021) have greater uncertainties associated with them and can provide high underestimates of extreme precipitation, leading to different scaling results from gauge observations. Yet even with gauge observations, factors such as data quality control, data measurement precision and statistical assumptions can severely affect estimated scaling rates severely at specific locations and moderately at regional/continental scales. These factors have not been adequately addressed in previous studies. Here we used extensively quality‐controlled hourly precipitation data from the GSDR data set for the CONUS region to identify differences in scaling rates arising from methodological choices. We summarize our results as follows:

1. We found a considerable difference in scaling rates over the CONUS using gauge data from NOAA/NCDC and quality‐controlled GSDR data. Scaling rates are higher for the quality‐controlled data, with all locations showing positive (median 6.2%/K) scaling, and a slightly higher proportion of gauges showing super‐CC scaling. This is important since previous scaling studies over the CONUS, such as Mishra et al. (2012) and Lepore et al. (2016), have used the NOAA/NCDC gauge data, thus underestimating the scaling rate.
2. Using two different data measurement precisions (0.25 and 2.54 mm) but for comparatively shorter records, we found substantially higher scaling rates for the higher measurement precision (0.25 mm: median 7.8%/K; 2.54 mm: median 6.6%/K), with the number of gauges showing super‐CC scaling also higher. This is an important result since all previous scaling studies over CONUS, for example, (Ali, Fowler, et al., 2021; Ali, Peleg, et al., 2021) have used the lower measurement precision, and thus underestimated the scaling rate in other global regions where a measurement precision of 0.1 mm is the norm. This may be a reason for the lower scaling rate in CONUS than in other parts of the world in some previous studies.
3. Using different statistical methods and assumptions to estimate scaling gives different scaling rates. We found the ZM method produced the highest scaling rates, with the QR method producing the lowest rates. The differences in scaling rates estimated from different statistical methods can be substantial. Pooling gauges together slightly reduces the estimated scaling rates compared to the estimated scaling rate at a single gauge.

Our results suggest that scaling rates are very sensitive to the choice of data and statistical assumptions and methods. One interesting future work could be to rank these three sources of uncertainty and find which one dominates the other. With the advancement in scaling studies, we, therefore, recommend that extensive quality checks and different statistical methods should be applied to observations to estimate robust scaling rates. In particular, the importance of seasonality cannot be overstated, with the ZM method appearing to provide a robust method for estimating scaling in different regions, including the Tropics (Ali, Fowler, et al., 2021). In addition, a consistent measurement precision should be used to remove any biases resulting from different measurement precisions in estimating scaling rates.

We note that our results here only consider the relation between precipitation intensities and temperature (SAT and DPT). These thermodynamic factors are important in the intensification of precipitation with global warming (Fowler, Ali., et al., 2021) but precipitation extremes may also change due to dynamic processes (as addressed in Pfahl et al., 2017). Further studies are needed which combine the role of thermodynamic and dynamic processes in intensifying precipitation extremes and assess how the relationship between precipitation intensities and day‐to‐day temperature variability from observations (apparent scaling) might be related to the climate scaling with global warming.

## Conflict of Interest

The authors declare no conflicts of interest relevant to this study.

## Supporting information

Supporting Information S1

## Data Availability

The hourly precipitation data (RAW‐DATA; identifier NCEI DSI‐3240) was downloaded from https://www.ncei.noaa.gov/access/metadata/landing-page/bin/iso?id=gov.noaa.ncdc:C00313;view=html. The authors downloaded the data files themselves and accompanying metadata from: https://data.nodc.noaa.gov/cgi-bin/iso?id=gov.noaa.ncdc:C00177 andhttps://doi.org/10.7289/V5NV9G8D. HadISD dewpoint temperature data is freely available at https://www.metoffice.gov.uk/hadobs/hadisd/.
