# Supplementary material for: Towards Quantifying the Uncertainty in Estimating Observed Scaling Rates
Source: Geophys Res Lett. 2022 Jun 18;49(12):e2022GL099138. doi: 10.1029/2022GL099138 (PMC9285755; doi:10.1029/2022GL099138)
Supplement: Supplementary file 1 — Supporting Information S1 [file GRL-49-0-s001.docx]

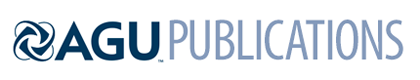


*Geophysical Research Letters*

Supporting Information for

**Towards quantifying the uncertainty in estimating observed scaling rates**

Haider Ali^1^, Hayley J. Fowler^1^, David Pritchard^1^, Geert Lenderink^2^, Stephen Blenkinsop^1^ and Elizabeth Lewis^1^

^1^School of Engineering, Newcastle University, Newcastle upon Tyne, UK

^2^Royal Netherlands Meteorological Institute, De Bilt, Netherlands

**Contents of this file**

Text S1 to S3

Figures S1 to S7

Table S1

**S1. Quality control of the GSDR data**

Lewis et al. (2021) developed a procedure to automate a quality control method on the GSDR data at the native hourly resolution and for daily aggregations. It includes 25 quality checks that follow World Meteorological Organisation (WMO) guidance, which recommends that rainfall data be subject to constraint, consistency, spike, rapid change, flat line (streak) and domain tests (WMO, 2019, 2018, 1986). The records are additionally checked for issues specific to sub-daily precipitation. For example, daily accumulations are sometimes incorrectly entered into hourly records, either as one (potentially non-zero) value every 24 hours or daily totals disaggregated uniformly across 24-hour periods. The GSDR-QC procedure uses the results of the quality checks in a simple “rule base” to exclude the most suspicious periods of data from gauge records. This step improves correspondence between GSDR records and neighbouring reference gauges in the Global Precipitation Climatology Centre (GPCC) database (Lewis et al., 2021). Given the inconsistent spatial and temporal coverage of the GSDR dataset, we use only PPT stations with a duration of at least 12 years (start and end year varying between 1979-2014), with missing hours in any given year at less than 20%.

**S2. Distance between PPT and DPT stations**

**
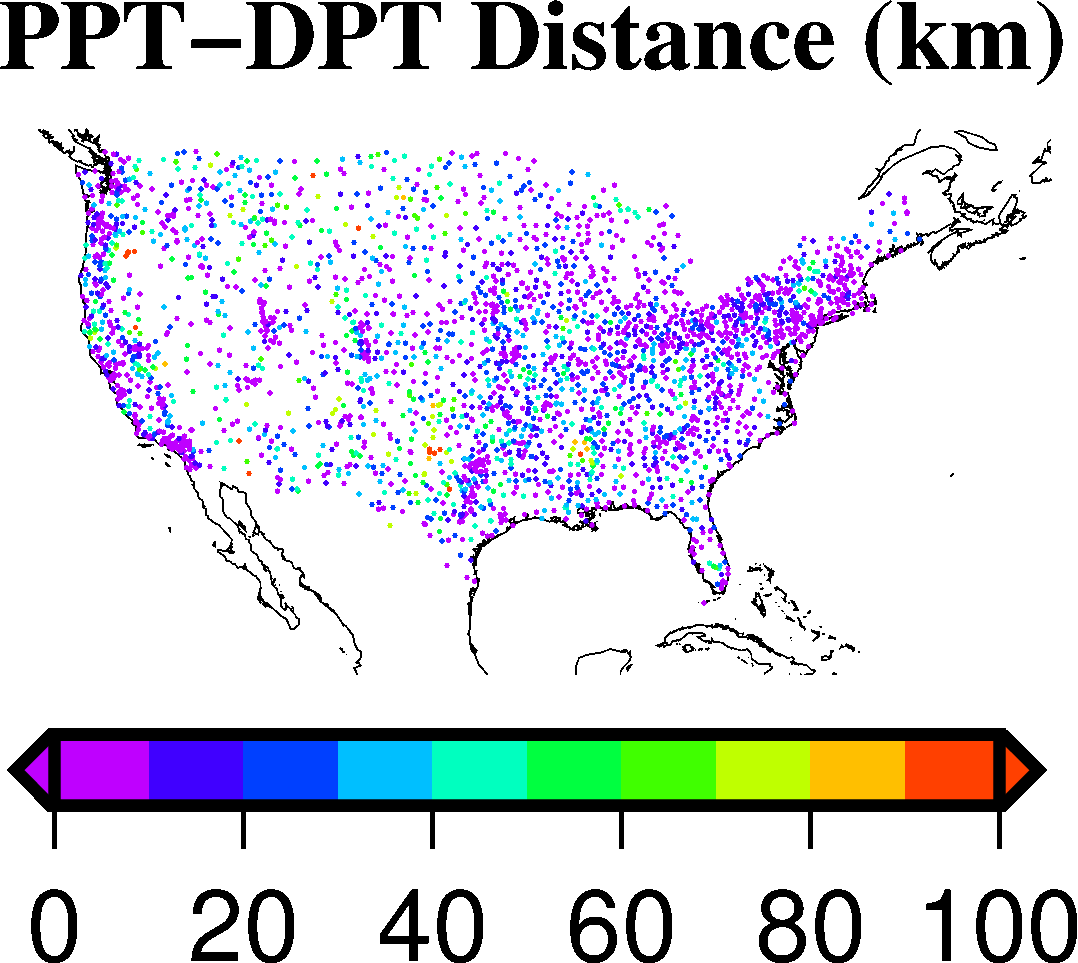
**

Figure S1: Distance (in kilometers) between precipitation (PPT) gauges from the GSDR dataset and the nearest dewpoint temperature (DPT) station from the HadISD dataset used to pair hourly PPT to daily DPT.

**S3. Examining the differences between scaling rates for RAW-DATA and GSDR-QC**

Lewis et al. (2021) analysed which quality checks in the GSDR-QC procedure lead to the removal of suspicious data in gauge records in the CONUS. This analysis showed that the vast majority of removals are related to implausibly long dry periods relative to the local climate. These periods are typical missing data – often over several years – that is inferred as zeros upon decoding the data format that can change scaling rates. The relevant GSDR-QC rules that identify this problem are R1, R9, R10 and R11 (discussed in Table S1). The rules use both neighbouring (GPCC) reference gauges and the ETCCDI CDD (http://etccdi.pacificclimate.org/list_27_indices.shtml) (continuous dry days) index in HadEX2 (Donat et al., 2013a) and GHCNDEX (Donat et al., 2013b) to identify these errors.

The GSDR-QC procedure also eliminates suspicious non-zero values in gauge records over the CONUS according to several other quality checks and rules. We identified 18 out of all 2905 gauges which have a scaling difference greater than 2%/K from the two versions of data. Here we discuss three stations in detail which have the highest scaling difference. The first example of this is shown in Figure S2 for the US_116605 (39.633^o^ N 87.7^o^ W) gauge. This gauge shows a scaling rate of -1.2%/K using RAW-DATA and a scaling rate of 5.6%/K using GSDR-QC data using binning method (Figure S3). Between the 23^rd^ and 30^th^ of April 1973, the gauge experienced some issues, before a period of missing data began in May 1973. These issues include two occurrences of precipitation at 508 mm/hour. These occurrences exceed the world record hourly precipitation (i.e. 401 mm/hr; WMO) and so are obviously in error. There is also a very low probability of such extreme hourly precipitation recurring so quickly and at the same value. The values are also exactly 100 times larger than the measurement resolution (2.54 mm) multiplied by two, which is indicative of possible unit or transcription error. The period also sees two occurrences of precipitation at exactly 251.46 mm/hour and one occurrence of 248.92 mm/hour.


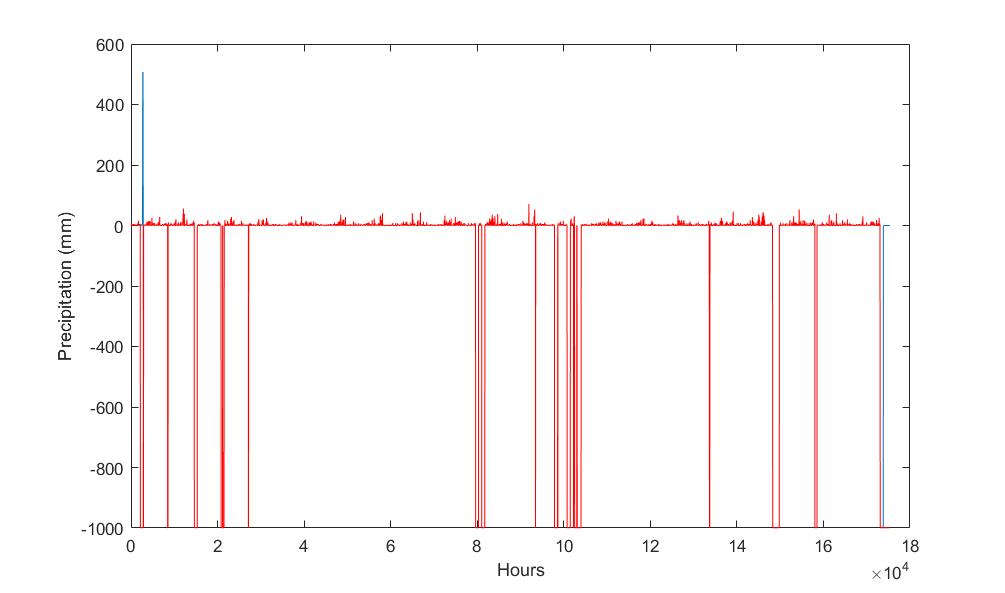


Supplemental Fig. S2: Time series of hourly rainfall for the US_116605 (39.633^o^ N 87.7^o^ W) from RAW-DATA (blue) and GSDR-QC (red).

Supplemental Fig. S3: Scaling curve for the US_116605 (39.633^o^ N 87.7^o^ W) from RAW-DATA (blue) and GSDR-QC (red). The same method as described in Fig.1 in the main paper was used to estimate scaling.

These extremes in the US_116605 data are flagged by some of the checks and rules within the GSDR-QC procedure. The two world record exceedances are flagged for removal by rule R5. Each extreme here is also flagged by rule R6, as hourly values above the record maximum daily precipitation for this location in the reference ETCCDI datasets (i.e. maximum Rx1day). In addition, the checks against neighbouring daily and monthly (GPCC) reference gauges both flag the period for removal using rules R8 and R11, respectively. These neighbouring gauges rules require agreement from multiple neighbours that a daily or monthly total stands out as being suspiciously large.

For the US gauges more generally, the mean (median) proportion of gauge series removed by GSDR-QC is 0.05% (0%), after excluding the rules removing suspiciously long dry periods. These percentages are based on the 3793 gauges with record periods over 20 years. The elimination of non-zero data is thus very small at most gauges. This might be partly because the US data are subject to prior quality control by the data providers, which helps to eliminate some erroneous extremes before the application of the GSDR-QC procedure. Most rules thus contribute only a very small amount to the overall data removal. For example, the world record hourly precipitation rule (R5) is invoked at only 18 of the 3793 gauges considered here. Similarly, the mean (median) proportion of a series flagged for removal by the checks on extremes against neighbouring (GPCC) daily gauges (R8) is 0.0004% (0%), with no removals at all for 84% of this sample of gauges. However, although suspicious extremes only comprise a small proportion of the US data series, they can have a disproportionate influence in various analyses, including precipitation-temperature scaling.

Notes on other example gauges:

- US_914229 (13.56^o^ N 144.84^o^ W): A high value was flagged by the monthly neighbours' check (QC20). However, this gauge was also adjusted during the manual review (i.e. after the application of the automated procedure) (Figure S4). Gauge time series were inspected when one or more of the quality flags or precipitation statistics appeared to stand out compared with neighbouring gauges. We did not record all details of this for each gauge, but it looks like the break in the record leads to some inhomogeneity. This is why the latter part of the series was removed after manual inspection. This gauge shows 0.27%/K scaling using RAW-DATA and 7.87%/K scaling using GSDR-QC data (Figure S5).


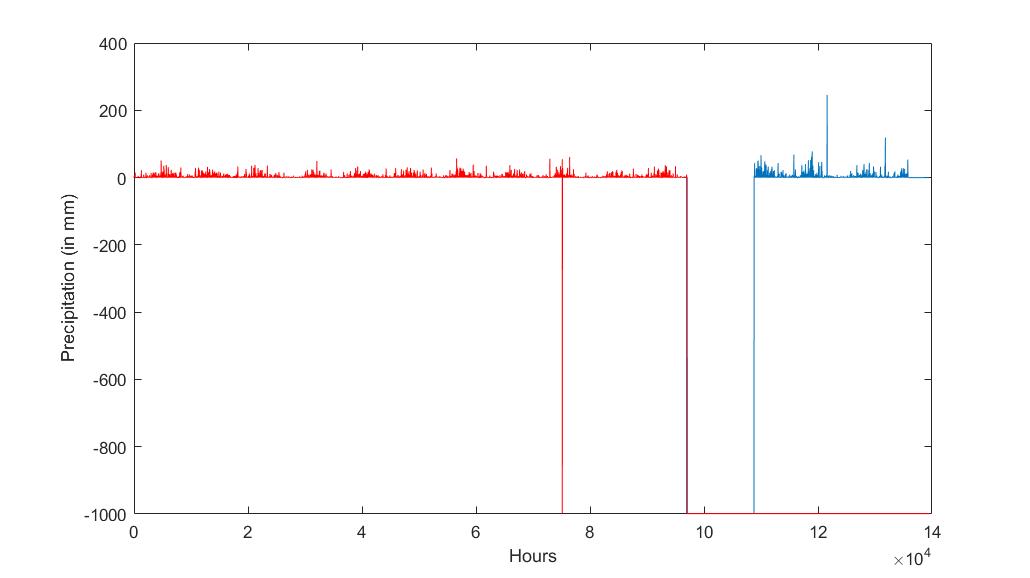


Supplemental Fig. S4: Time series of hourly rainfall for the US_914229 (13.56^o^ N 144.84^o^ W) from RAW-DATA (blue) and GSDR-QC (red).

Supplemental Fig. S5: Scaling curve for the US_914229 (13.56^o^ N 144.84^o^ W) from RAW-DATA (blue) and GSDR-QC (red). The same method as described in Fig1 in the main paper was used to estimate scaling.

- US_020768 (31.433^o^ N 109.917^o^ W): The error here is effectively a streak of repeated values (Figure S6). This is normally picked up in QC15 and R4, but in this case, there is an inconsistent time difference between each repetition, which means R4 does not identify it. However, the monthly neighbours' check/rule (R11) identifies this part of the series as erroneous, because it leads to an excessive monthly total relative to multiple neighbouring gauges. So this is an example of how multiple checks can help identify issues missed by the specific implementation of another check. (Similarly, the US_116605 example given in the text above also shows how multiple checks can agree that a period of data should be removed). This gauge shows 3.75%/K scaling using RAW-DATA and 12.8%/K scaling using GSDR-QC data (Figure S7).


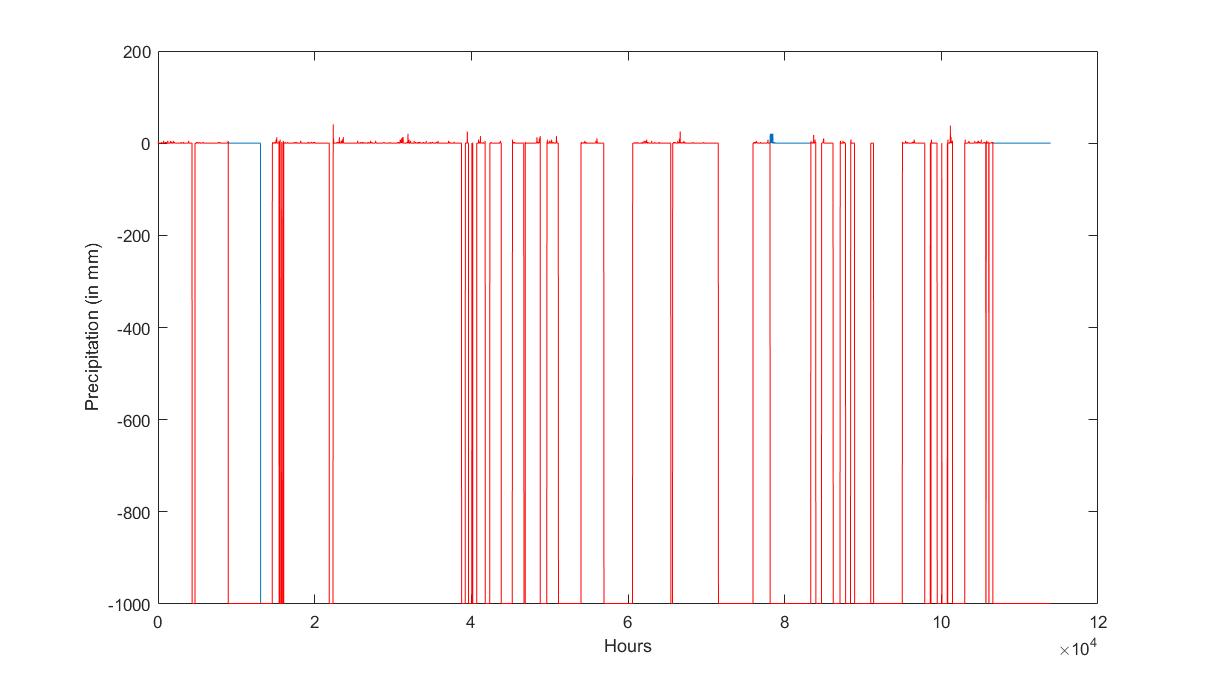


Supplemental Fig. S6: Time series of hourly rainfall for the US_020768 (31.433^o^ N 109.917^o^ W) from RAW-DATA (blue) and GSDR-QC (red).

Supplemental Fig. S7: Scaling curve for the US_020768 (31.433^o^ N 109.917^o^ W) from RAW-DATA (blue) and GSDR-QC (red). The same method as described in Fig1 was used to estimate scaling.

Table S1: The important GSDR Quality Control checks that remove suspicious data (derived from Table 3 in Lewis et al., 2021).

| **Rule** | **QC Flags** | **Description** |
| --- | --- | --- |
| R1 | K-largest (QC2) - k10 = 0 | 10 largest events in a year all equal zero – excludes full year |
| R9 | Hourly neighbours (dry) (QC19) = 3 & CDD (QC12) > 0 | Excessive dry spells according to hourly neighbours and/or ETCCDI CDD. In the absence of neighbours, the CDD flag is relied upon |
| R10 | Daily neighbours (dry) (QC18) = 3 & CDD (QC12) > 0 | Excessive dry spells according to daily neighbours and/or ETCCDI CDD. In the absence of neighbours, the CDD flag is relied upon |
| R11 | Monthly neighbours (QC20) = 4 or 5 | Monthly neighbours discrepancy (i.e. for very high totals relative to all neighbours) |

**References:**

Donat, M. G., Alexander, L. V., Yang, H., Durre, I., Vose, R., Dunn, R. J., ... & Kitching, S. (2013a). Updated analyses of temperature and precipitation extreme indices since the beginning of the twentieth century: The HadEX2 dataset. *Journal of Geophysical Research: Atmospheres*, *118*(5), 2098-2118.

Donat, M. G., Alexander, L. V., Yang, H., Durre, I., Vose, R., & Caesar, J. (2013b). Global land-based datasets for monitoring climatic extremes. *Bulletin of the American Meteorological Society*, *94*(7), 997-1006.

Lewis, E., Pritchard, D., Villalobos-Herrera, R., Blenkinsop, S., McClean, F., Guerreiro, S., ... & Fowler, H. J. (2021). Quality control of a global hourly rainfall dataset. *Environmental Modelling & Software*, *144*, 105169.
